# Assessing metacognitive beliefs about worry: validation of German versions of the *Why Worry Scale II* and the *Consequences of Worry Scale*

**DOI:** 10.7717/peerj.5177

**Published:** 2018-07-13

**Authors:** Carolin Thielsch, Tanja Andor, Thomas Ehring

**Affiliations:** 1 Department of Psychology, University of Münster, Münster, Germany; 2 Department of Psychology, LMU, Munich, Germany

**Keywords:** Metacognitions, Metacognitive beliefs, Worry, Generalized anxiety disorder, Meta-worry

## Abstract

**Background:**

Metacognitive beliefs have been proposed to play a key role in initiating and maintaining worry. The *Why Worry-Scale-II (WW-II)* and *Consequences of Worry Scale (COWS)* are self-report questionnaires assessing positive and negative metacognitive beliefs. The main goal of this study was to validate German versions of these two questionnaires.

**Method:**

*N* = 603 participants completed a questionnaire battery, including the two self-report measures of metacognitive beliefs. We conducted confirmatory factor analyses, calculated internal consistencies, and examined convergent and divergent validity. In addition, the questionnaires’ power in predicting worry, repetitive negative thinking (RNT) and generalized anxiety disorder (GAD) symptoms were investigated.

**Results:**

The factor structure of the original versions could be replicated for both measures. Furthermore, the translated questionnaires demonstrated excellent internal consistency and evidence of convergent and divergent validity. Importantly they also possessed predictive power in explaining worry, RNT and GAD symptoms, even over and above the Metacognitions Questionnaire-30 (MCQ-30) as the current gold standard.

**Conclusions:**

Overall, our findings suggest that the WW-II and COWS show solid psychometric properties and are useful in measuring metacognitive beliefs independently from the MCQ-30.

## Introduction

Worry is a repetitive thought activity that ranges from an everyday phenomenon to an excessive and seemingly uncontrollable manifestation. Excessive uncontrollable worry is the key characteristic of generalized anxiety disorder (GAD; APA, 2013), while worry also is an everyday phenomenon and most researchers conceptualize it on a continuum, with quantitative rather than qualitative differences between worry in non-clinical vs. clinical samples (Ehring & Watkins, 2008). Current cognitive models of dysfunctional worry and GAD suggest that metacognitive beliefs (“thoughts about thoughts”) are crucial in initiating and maintaining worrisome thinking (Behar et al., 2009) and play an important role across psychopathologies (Sun, Zhu & So, 2017).

In his *Metacognitive Model*, Wells (1995) suggests that *positive metacognitions* (e.g., “Worry helps to cope.”) initiate worry in response to internal or external cues (e.g., “I might fail the test.”). Worrying then activates *negative metacognitions* (e.g., “Worry is uncontrollable.”), leading to meta-worry or ‘worry about worry’ (e.g., “If I start worrying now, I will not be able to stop.”), which again evokes engagement in thought control. Paradoxically, thought suppression leads to the maintenance of worry, strengthens the negative metacognitive belief and causes negative emotional responses. Thus, evidence shows that worry maintains the problem instead of reducing it while having an alarming rather than calming effect.

Likewise, the *Intolerance of Uncertainty Model* (Dugas et al., 1998) presents *positive metacognitions* as a key construct. Individuals with high intolerance of uncertainty are proposed to experience stress in response to ambiguous situational cues, leading to the activation of *positive beliefs about worry* (i.e., beliefs in worry as an aid to problem solving and motivation, as protection from negative emotions, as a positive personality trait, and as a possibility to directly alter events; Francis & Dugas, 2004), whereby worrisome thinking is initiated.

In line with these theoretical conceptualizations, a large number of studies have revealed significant associations between metacognitions and worry (for a review, see Behar et al., 2009; Dugas & Robichaud, 2012; Wells, 2004). Negative metacognitions (particularly those related to uncontrollability and danger of worrying) were found to show a very close link to worrisome thinking, while results concerning the predictive value of positive metacognitions are inconsistent (Cartwright-Hatton & Wells, 1997; Davis & Valentiner, 2000; Ruscio & Borkovec, 2004; Thielsch, Andor & Ehring, 2015a; Thielsch, Andor & Ehring, 2015b).

Most studies to date have used the *Metacognitions Questionnaire* (*MCQ*; Cartwright-Hatton & Wells, 1997; short form MCQ-30 Wells & Cartwright-Hatton, 2004), which is often regarded as the gold standard measure of metacognitive beliefs about worry and is a well-validated measure (Wells & Cartwright-Hatton, 2004). However, the MCQ-30 can be criticized on at least two grounds: first, it assesses positive and negative metacognitive beliefs about worry unidimensionally and does not capture subdimensions of them (e.g., subscales of positive metacognitions as postulated by Dugas & Robichaud, 2012). In addition, it has been argued that predicting excessive worrying by the negative metacognition-subscale referring to uncontrollability and danger may be circular, as there is a conceptual overlap between the predictor (i.e., the belief that worry is uncontrollable) and the criterion (i.e., self-report of uncontrollable worry)^1^ 1The MCQ-65 (8 out of 16 items measuring negative beliefs directly refer to the uncontrollability of worrying) and MCQ-30 (three out of six items aim at capturing the perceived lack of control: “My worrying thoughts persist, no matter how I try to stop them”, “When I start worrying I cannot stop”, “I cannot ignore my worrying thoughts”) show overlap with the PSWQ (example item “Once I start worrying, I cannot stop.”) that is often used as a criterion.(Behar et al., 2009; Gerlach, Andor & Patzelt, 2008; Wells, 2005).

Due to these limitations, it appears necessary to additionally use questionnaire measures beyond the MCQ-30 when investigating the relationship between metacognitive beliefs and worry. The *Why Worry Scale II* (*WW*: Freeston et al., 1994; *WW-II*; Gosselin et al., 2003) and the *Consequences of Worry Scale* (*COWS*; Davey, Tallis & Capuzzo, 1996) are alternative questionnaire measures assessing metacognitions about worry. The WW-II specifies positive beliefs about worry on five subscales, while the COWS is comprised of items referring to both negative (three subscales) as well as positive consequences (two subscales) about worrying. Importantly, these measures can be expected to overcome some of the problems related to an exclusive use of the MCQ-30: first, both questionnaires examine different facets of positive and negative metacognitions. In addition, while the MCQ-30 subscale measuring negative beliefs about worry is mainly focused on the uncontrollability of worry, the COWS assesses a broad range of negative metacognitions, including worrying disrupting effective performance, exaggerating the problem and causing emotional discomfort.^2^ 2In contrast to the MCQ, the COWS directly refers to uncontrollability with only one out of 17 items (“Deep down I know I do not need to worry that much but I can’t help it.”).Therefore, it can be argued that the questionnaire shows less criterion contamination with measures of excessive worrying than the MCQ-30.

This study aimed at validating German versions of the WW-II and the COWS. The questionnaires were translated into German, followed by a test of their factor structure and a psychometric evaluation. We expected to find the proposed five-factor structures for the translated versions as well as evidence for good psychometric attributes that have been reported for the original questionnaires (Covin, Dozois & Westra, 2008; Hebert et al., 2014). Additionally, we expected a diagnostic value in the sense of variance explanation in worry and related phenomena based on the WW-II and COWS—also over and above the MCQ-30.

## Method

### Participants

Participants for the validation study were invited via the online-panel PsyWeb (https://psyweb.uni-muenster.de/). Inclusion criteria were German as the first language and at least 18 years of age.

Eight hundred eighty panel members started the study, *n* = 274 did not complete the study and *n* = 3 withdrew their consent for their data being analyzed at the end of the study. Therefore, 603 participants (69% female) between 18 and 84 years of age (*M*= 46.40, *SD* = 13.94) were included in the analyses (education level reported most frequently: 64% had completed an academic type of high school; employment status reported most frequently: 61% classified themselves as currently employed).

The average PSWQ score (*M* = 40.66, *SD* = 7.86: below the cut-off score 62 for non-clinical samples; Behar et al., 2003) and the average GAD-Q-IV score (*M* = 3.78, *SD* = 3.77: below the cut-off score of 7.67; Moore et al., 2014) overall reflect the non-clinical nature of the sample.

### Measures

The *Penn State Worry Questionnaire* (*PSWQ*; Meyer et al., 1990; German version: Stöber, 1995) measures worrisome thinking using 16 items (e.g.; “I am always worrying about something”) and a five-point Likert scale, ranging from “not at all typical of me” to “very typical of me”. High internal consistency and good test-retest reliability have been reported (PSWQ; Fresco et al., 2002; Stöber, 1995; in this sample *α* = .74).

The *Perseverative Thinking Questionnaire* (PTQ; Ehring et al., 2011) aims at capturing the process of recurring thinking. Independent of a specific type of mental illness, it focuses on repetitive negative thinking (RNT) using 15 items with regard to the key characteristics of the phenomenon (repetitiveness; intrusiveness; difficulties with disengagement) and two further features (unproductiveness of RNT; mental capacity captured by RNT) that have to be rated on a five-point Likert scale ranging from “never” to “always”. The measure has been shown to possess good psychometric properties (Ehring et al., 2011; in this sample *α* = .96).

The *Generalized Anxiety Disorder Questionnaire-IV* (GAD-Q-IV; Newman et al., 2002) examines the DSM-IV criteria of GAD with nine items measuring excessiveness and uncontrollability of worry (German version: Hoyer). The questionnaire consists of five dichotomous items assessing the intensity, perceived uncontrollability and specific triggers of worries, a list of the most frequent worry topics, while afterwards the presence or absence of six related physical symptoms has to be reported and ratings of functional impairment and subjective distress (nine-point Likert scales ranging from “none” to “very severe”) are required. High test-retest reliability, diagnostic sensitivity and specificity, as well as convergent and discriminant validity have been reported (Newman et al., 2002; Robinson, Klenck & Norton, 2010). Moore et al. (2014) present a total score of 7.67 as an appropriate cut-off score to screen for GAD in primary care.

The *Metacognitions Questionnaire* (MCQ; Cartwright-Hatton & Wells, 1997; short form Wells & Cartwright-Hatton, 2004; German version Arndt et al., 2011a; Arndt et al., 2011b) uses 65 items (original version), respectively 30 items (short form) to measure metacognitive beliefs about worry (example items: “My worrying is dangerous for me.”, “Worrying helps me to solve problems.”). We used the MCQ-30, which comprises five scales, of which two specifically refer to negative and positive metacognitive beliefs initiating type 1 and type 2 worry (negative beliefs about uncontrollability and danger of worrisome thoughts; positive beliefs about worry).^3^ 3A third scale named “negative beliefs about the need to control thoughts” captures metacognitive beliefs about the coping strategy of thought control, a phenomenon that in the Metacognitive Model (Wells, 1997; Wells, 2005) is conceptually separated from meta-cognitive beliefs that play a role in initiating type 1 and type 2 worry. Two other subscales measure “cognitive confidence” and “cognitive self-consciousness”. It has been shown to possess good internal consistency and convergent validity as well as good test-retest reliability (Arndt et al., 2011a; Arndt et al., 2011b; Möbius & Hoyer, 2003; Wells & Cartwright-Hatton, 2004; in this sample *α* = .88 for all 30 items).

The *Why Worry Scale II* (WW: Freeston et al., 1994; WW-II; Gosselin et al., 2003) is a 25-item self-report questionnaire to measure positive beliefs about worry (e.g., “If I did not worry, I would be careless and irresponsible.”) on five subscales (worry facilitates problem solving; worry enhances motivation; worry protects against negative emotions; worry prevents negative outcomes; worry reflects a positive personality trait). Items have to be rated on a five-point Likert scale ranging from “not at all true” to “absolutely true”. Excellent internal consistency and good test–retest reliability as well as a predictive power for worry severity could be demonstrated for the English version (Hebert et al., 2014). With permission from the original authors of the measure, the questionnaire was translated into German by the authors of this study.

The *Consequences of Worry Scale* (*COWS*; Davey, Tallis & Capuzzo, 1996) comprises 29 items capturing negative beliefs (e.g., “Worry gets me worked up.”) and positive beliefs (e.g., “Worrying stimulates me.”) about the consequences of worrying. The items regress on two subscales (negative beliefs, positive beliefs) and five factors (worrying disrupts effective performance, worrying exaggerates the problem, worrying causes emotional discomfort, worry motivates, worry helps analytic thinking). Responses are rated on a five-point Likert scale. Good internal reliability could be reported for the subscales of the original version (Covin, Dozois & Westra, 2008; Davey, Tallis & Capuzzo, 1996). The questionnaire was translated into German by the authors of this study with permission of the original author.

### Procedure

The German versions were generated following standard procedures (Schmitt & Eid, 2007). The questionnaires were first translated from English to German by the authors. Next, a bilingual psychologist who was unaware of the original questionnaires back-translated the material. In case of inconsistencies, a consensus-finding process was initiated. Afterwards a group of psychology students checked the translated versions for comprehensibility. Participants of the validation study completed all measures online. Before completing the questionnaires, written informed consent was acquired.

#### Ethics approval and consent to participate

All procedures were performed in compliance with relevant laws and institutional guidelines following the Helsinki Declaration of 1975, as revised in 2013. We observed privacy rights, obtained written informed consent from all individual participants included in the study and disclosed any conflicts of interests with study participants.

### Statistical analyses

Descriptive parameters, internal consistencies, correlational relationships and regression analyses were computed using SPSS (version 22; IBM, Armonk, NY, USA).

In order to examine the factor structure of the questionnaires, confirmatory factor analyses with diagonally weighted least squares (DWLS) estimation (Mindrila, 2010) were conducted using R Statistical Software (R Development Core Team, 2008).

## Results

### Confirmatory factor analyses

Two confirmatory factor analyses (CFA) were conducted to test the factor structures of the WW-II and COWS. On the basis of the theoretical and empirical literature, more precisely, based on the results of the factor analyses in the original papers (Davey, Tallis & Capuzzo, 1996; Gosselin et al., 2003), five-factor structures were assumed for both questionnaires. With factor loadings greater than 0.35 (see Tables 1 and 2; c.f., Field, 2007), all items loaded significantly on the corresponding factor.^4^ 4Examining modification indices for the CFA revealed that COWS item 8 shows a similar amount of loading on factor II as it does regarding factor I, so it might not be possible to unambiguously ascribe it to one or the other subscale and one might consider deleting it when using the questionnaire in future research if that is seen as problematic.

**Table 1:** Confirmatory factor analysis: factor loadings of the WW-II Items (*n* = 603).

		I	II	III	IV	*V*	
1.	Wenn ich mir keine Sorgen machen würde, wäre ich nachlässig und verantwortungslos. [If I did not worry, I would be careless and irresponsible.]					0.75	
2.	Wenn ich mir Sorgen mache, werde ich weniger beunruhigt sein, wenn unvorhergesehene Ereignisse eintreten. [If I worry, I will be less disturbed when unforeseen events occur.]			0.72		
3.	Ich mache mir Sorgen, um zu wissen, was ich tun muss. [I worry in order to know what to do.]	0.78				
4.	Wenn ich mir im Voraus Sorgen mache, werde ich weniger enttäuscht sein, wenn etwas Ernstes passiert. [If I worry in advance, I will be less disappointed if something serious occurs.]			0.72		
5.	Die Tatsache, dass ich mir Sorgen mache, hilft mir bei der Planung von Handlungen zur Lösung eines Problems. [The fact that I worry helps me plan my actions to solve a problem.]	0.86				
6.	Sich Sorgen zu machen an sich kann verhindern, dass Misserfolge auftreten. [The act of worrying itself can prevent mishaps from occurring.]				0.74	
7.	Wenn ich mir keine Sorgen machen würde, würde mich das zu einer nachlässigen Person machen. [If I did not worry, it would make me a negligent person.]					0.81	
8.	Dadurch, dass ich mir Sorgen mache, nehme ich letztendlich die Arbeit in Angriff, die ich zu erledigen habe. [It is by worrying that I finally undertake the work that I must do.]		0.80			
9.	Ich mache mir Sorgen, weil ich denke, dass es mir dabei hilft, eine Lösung für mein Problem zu finden. [I worry because I think it can help me find a solution to my problem.]	0.84				
10.	Die Tatsache, dass ich mir Sorgen mache zeigt, dass ich eine pflichtbewusste Person bin. [The fact that I worry shows that I am a person who takes care of their affairs.]					0.85	
11.	Zu viel über positive Ereignisse nachzudenken kann verhindern, dass diese eintreten. [Thinking too much about positive things can prevent them from occurring.]				0.47	
12.	Die Tatsache, dass ich mir Sorgen mache zeigt, dass ich eine umsichtige Person bin. [The fact that I worry confirms that I am a prudent person.]					0.88	
13.	Wenn ein Unglück eintritt, werde ich mich weniger verantwortlich dafür fühlen, wenn ich mir vorher Sorgen darüber gemacht habe. [If misfortune comes, I will feel less responsible if I have been worrying about it beforehand.]			0.76		
14.	Dadurch, dass ich mir Sorgen mache, kann ich einen besseren Weg finden, Dinge zu erledigen. [By worrying, I can find a better way to do things.]	0.86				
15.	Mir Sorgen zu machen aktiviert mich und macht mich effektiver. [Worrying stimulates me and makes me more effective.]		0.87			
16.	Die Tatsache, dass ich mir Sorgen mache, spornt mich zu Taten an. [The fact that I worry incites me to act.]		0.89			
17.	Sich Sorgen zu machen an sich reduziert das Risiko, dass etwas Schlimmes passiert. [The act of worrying itself reduces the risk that something serious will occur.]				0.74	
18.	Dadurch, dass ich mir Sorgen mache, nehme ich Dinge in Angriff, für die ich mich sonst nicht entscheiden würde. [By worrying, I do certain things which I would not decide to do otherwise.]		0.78			
19.	Die Tatsache, dass ich mir Sorgen mache, motiviert mich, die Dinge zu erledigen, die ich tun muss. [The fact that I worry motivates me to do the things I must do.]		0.85			
20.	Meine Sorgen selbst können die Risiken von Gefahr reduzieren. [My worries can, by themselves, reduce the risks of danger.]				0.74	
21.	Wenn ich mir weniger Sorgen mache, verringere ich die Wahrscheinlichkeit, die beste Lösung zu finden. [If I worry less, I decrease my chances of finding the best solution.]	0.63				
22.	Die Tatsache, dass ich mir Sorgen mache, wird es mir ermöglichen, mich weniger schuldig zu fühlen, wenn etwas Schlimmes passiert. [The fact that I worry will allow me to feel less guilty if something serious occurs.]			0.76		
23.	Wenn ich mir Sorgen mache, werde ich weniger unglücklich sein, wenn ein negatives Ereignis eintritt. [If I worry, I will be less unhappy when a negative event occurs.]			0.73		
24.	Man kann Unglück dadurch anziehen, dass man sich keine Sorgen macht. [By not worrying, one can attract misfortune.]				0.49	
25.	Die Tatsache, dass ich mir Sorgen mache, zeigt, dass ich ein guter Mensch bin. [The fact that I worry shows that I am a good person.]					0.65	

**Notes.**

Factor loading are significant with *p* < 0.001.

Subscales WW-II: I worry facilitates problem solving, II worry enhances motivation, III worry protects against negative emotions, IV worry prevents negative outcomes, V worry reflects a positive personality trait.

**Table 2:** Confirmatory factor analysis: factor loadings of the COWS items (*n* = 603).

		I	II	III	IV	V
1.	Mir Sorgen zu machen erhöht meine Angst und verringert dadurch meine Leistungsfähigkeit. [Worrying increases my anxiety and so decreases my performance.]	0.84				
2.	Wenn ich mir Sorgen mache, hindert es mich daran, tatkräftig zu handeln. [When I worry it stops me taking decisive action.]	0.88				
3.	Mir Sorgen zu machen deprimiert mich und macht es schwieriger, mich zu konzentrieren und Dinge zu erledigen. [Worrying makes me depressed and therefore makes it harder to concentrate and get on with things.]	0.88				
4.	Mein Sorgen führt dazu, dass ich mich auf die falschen Dinge konzentriere. [Worry makes me focus on the wrong things.]	0.84				
5.	Mir Sorgen zu machen verzerrt meine Sicht auf das Problem, das ich habe, und daher kann ich es nicht lösen. [Worrying distorts the problem I have and so I am unable to solve it.]	0.84				
6.	Mir Sorgen zu machen führt dazu, dass ich eine pessimistische und schicksalsergebene Perspektive einnehme. [Worrying gives me a pessimistic and fatalistic outlook.]	0.78				
7.	Durch das Sorgen habe ich weniger Energie, um auf die Ereignisse zu reagieren, wegen der ich mich sorge. [Worrying weakens me by affecting my levels of energy in response to those events that worry me.]	0.88				
8.	Mir Sorgen zu machen führt dazu, dass ich irrational werde. [Worrying makes me irrational.]	0.79				
9.	Ich werde paranoid wenn ich mir Sorgen mache. [I become paranoid when I worry.]		0.65			
10.	Mir Sorgen zu machen verhindert, dass ich klar denken kann. [Worrying stops me from thinking straight.]		0.83			
11.	Eigentlich weißich, dass ich mir nicht so viele Sorgen zu machen brauche, aber ich kann es nicht sein lassen. [Deep down I know I do not need to worry that much but I can’t help it.]		0.48			
12.	Probleme vergrößern sich, wenn ich mich zu viel mit ihnen beschäftige. [Problems are magnified when I dwell on them.]		0.65			
13.	Mir Sorgen zu machen hindert mich daran, mit bestimmten Situationen umzugehen. [Worrying stops me dealing with certain situations.]		0.81			
14.	Sorgen löst bei mir Stress aus. [Worry causes me stress.]			0.85		
15.	Mir Sorgen zu machen vergrößert meine Angst. [Worrying increases my anxiety.]			0.88		
16.	Mir Sorgen zu machen regt mich auf. [Worrying gets me worked up.]			0.84		
17.	Mich zu sorgen macht mich angespannt und reizbar. [Worrying makes me tense and irritable.]			0.85		
18.	Mir Sorgen zu machen spornt mich an. [Worrying acts as a stimulant.]				0.74	
19.	Mir Sorgen zu machen fordert mich heraus und motiviert mich; ohne meine Sorgen würde ich in meinem Leben nicht viel erreichen. [Worrying challenges and motivates me, without them I would not achieve much in life.]				0.83	
20.	Um etwas geschafft zu bekommen, muss ich mir darüber Sorgen machen. [In order to get something done I have to worry about it.]				0.72	
21.	Mir Sorgen zu machen verbessert meine Wahrnehmung und somit auch meine Leistung. [Worrying increases my awareness thus increasing my performance.]				0.85	
22.	Mir Sorgen zu machen lässt mich Dinge schaffen, da sich mein Adrenalinspiegel erhöht. [Worrying makes me do things by increasing my adrenaline levels.]				0.78	
23.	Mir Sorgen zu machen lässt Gedanken klarer werden und verbessert die Konzentration. [Worrying clarifies thoughts and concentration.]				0.82	
24.	Mir Sorgen zu machen führt dazu, dass ich über mein Leben nachdenke, da Fragen auftauchen, die ich mir üblicherweise nicht stelle, wenn ich glücklich bin. [Worrying makes me reflect on life by asking questions I might not usually ask when happy.]					0.61	
25.	Mir Sorgen zu machen gibt mir die Möglichkeit, Situationen zu analysieren und die Vor-und Nachteile abzuwägen. [Worrying gives me the opportunity to analyze situations and work out the pros and cons.]					0.86	
26.	Mir Sorgen zu machen setzt einen Prozess zur Vorbereitung auf neue Situationen in Gang. [Worrying starts off as a process of preparing me to meet new situations.]					0.85	
27.	Mir Sorgen zu machen erlaubt es mir, mich mit dem Schlimmsten zu beschäftigen, und bringt Erleichterung, wenn dies ausbleibt. [Worrying allows me to work through the worst that can happen, so when it doesn’t happen things are better.]					0.60	
28.	Sich Sorgen zu machen bekräftigt die Wichtigkeit eines Problems und lässt mich dadurch verschiedene Möglichkeiten abwägen. [Worrying adds concern to the problem and as such leads me to explore different possibilities.]					0.80	
29.	Dadurch, dass ich mir Sorgen mache, teile ich mir meine Zeit besser ein; wenn ich das beibehalte, fühle ich mich dadurch besser. [By worrying, I reorganize and plan my time better - if I stick to it, it makes me feel better.]					0.57	


**Notes.**

Factor loading are significant with *p* < 0.001.

Subscales COWS: COWS-negative: I worrying disrupts effective performance, II worrying exaggerates the problem, III worrying causes emotional discomfort; COWS-positive: IV worry motivates, V worry helps analytic thinking.

The individual CFAs altogether revealed a good fit to the structure. When modeling large sample sizes, the chi-square test is considered very strict, so the unwanted significant results (WW-II: *chi-square* = 666.428; *df* = 265; *p* < .001; COWS: *chi-square* = 1363.271; *df* = 367; *p* < .001) seem tolerable and alternative measures of approximate fit were brought in. The RMSEA (WW-II: *RMSEA* = .050; COWS: *RMSEA* = .067) provides a good fit, as both values do not exceed the suggested maximum of .07 (Steiger, 2007). As for the SRMR, values as high as .08 are deemed acceptable (Hu & Bentler, 1999), which was slightly exceeded by the COWS (*SRMR* = .063 for the Why Worry-II and .087 for the COWS). CFI and NFI as goodness-of-fit indexes met the proposed threshold of .95 (WW-II: *CFI* = .988, *NFI* = .981; COWS: *CFI* = .977, *NFI* = .968) (Hu & Bentler, 1999).

### Internal consistency

Internal consistencies for all scales were good to excellent (WW-II: .77 < *α* < .92; COWS: .81 < *α* < .95; see Table 3).

**Table 3:** Alpha coefficients.

Questionnaire	Subscale	Number of Items	Cronbach’s alpha (*α*)
Why Worry II	I) problem solving	5	.89
	II) motivation	5	.92
	III) protection	5	.85
	IV) prevention	5	.77
	V) positive trait	5	.89
	Total score	25	.95
COWS	I) disruption	8	.95
	II) problem exaggeration	5	.81
	III) emotional discomfort	4	.92
	COWS-neg	17	.96
	IV) motivation	6	.91
	VI) analytic thinking	6	.86
	COWS-pos	12	.92

**Notes.**

Subscales WW-II: I worry facilitates problem solving, II worry enhances motivation, III worry protects against negative emotions, IV worry prevents negative outcomes, V worry reflects a positive personality trait.

COWS-negative: I worrying disrupts effective performance, II worrying exaggerates the problem, III worrying causes emotional discomfort.

COWS-positive: IV worry motivates, V worry helps analytic thinking; for means and standard deviations see Table 5.

### Convergent and divergent validity

Inter-correlations were calculated for the WW-II/COWS subscales (Table 4) and for all measures used in this study (Table 5).

**Table 4:** Inter-Correlations of the Why Worry-II and COWS scores.

	Measure	1.	2.	3.	4.	5.	6.	7.	8.	9.	10.	11.	12.	13.
Why Worry II	1. I) problem solving	–	.76^***^	.66^***^	.66^***^	.67^***^	.89^***^	−.17^***^	−.02	−.01	−.10^*^	.68^***^	.68^***^	.74^***^
	2. II) motivation		–	.52^***^	.57^***^	.60^***^	.83^***^	−.26^***^	−.10^*^	−.06	−.18^***^	.73^***^	.59^***^	.72^***^
	3. III) protection			–	.66^***^	.63^***^	.81^***^	.05	.21^***^	.19^***^	.13^**^	.44^***^	.52^***^	.53^***^
	4. IV) prevention				–	.71^***^	.83^***^	−.04	.10^*^	.06	.02	.50^***^	.48^***^	.54^***^
	5. V) positive trait					–	.86^***^	−.05	.10^*^	.06	.02	.50^***^	.54^***^	.57^***^
	6. WW II total						–	−.12^**^	.06	.053	−.03	.682^**^	.67^***^	.74^***^
COWS	7. I) disruption							–	.82^***^	.73^***^	.96^***^	−.23^***^	−.11^**^	−.19^***^
	8. II) problem exaggeration								–	.76^***^	.92^***^	−.08	.03	−.02
	9. III) emotional discomfort									–	.87^***^	−.08^*^	.05	−.01
	10. COWS-neg total										–	−.17^***^	−.04	−.11^**^
	11. IV) motivation											–	.67^***^	.90^***^
	12. V) analytic thinking												–	.92^***^
	13. COWS-pos total													–

**Notes.**

^∗^*p* < .05, ^∗∗^*p* < .01, ^∗∗∗^*p* < .001 (two-sided).

Subscales WW-II: I worry facilitates problem solving, II worry enhances motivation, III worry protects against negative emotions, IV worry prevents negative outcomes, V worry reflects a positive personality trait.

COWS-negative: I worrying disrupts effective performance, II worrying exaggerates the problem, III worrying causes emotional discomfort.

COWS-positive: IV worry motivates, V worry helps analytic thinking.

**Table 5:** Inter-correlations, means and standard deviations of the study measures.

	Measure	MCQ-neg	MCQ-pos	PSWQ	PTQ	GAD-Q-IV	M	SD
Why Worry II	I) problem solving	.17^***^	.80^***^	.29^***^	.22^***^	.16^***^	11.58	4.68
	II) motivation	.08^*^	.67^***^	.24^***^	.12^**^	.09^*^	11.70	5.02
	III) protection	.38^***^	.53^***^	.45^***^	.38^***^	.34^***^	10.25	4.36
	IV) prevention	.28^***^	.52^***^	.33^***^	.28^***^	.24^***^	8.63	3.56
	V) positive trait	.27^***^	.56^***^	.35^***^	.29^***^	.27^***^	11.09	4.79
	WW II total	.27^***^	.74^***^	.39^***^	.30^***^	.26^***^	53.25	18.94
COWS	I) disruption	.51^***^	−.22^***^	.39^***^	.51^***^	.41^***^	21.90	9.43
	II) problem exaggeration	.64^***^	−0.07	.51^***^	.63^***^	.52^***^	12.13	4.83
	III) emotional discomfort	.60^***^	−0.06	.49^***^	.59^***^	.54^***^	12.69	4.78
	COWS-neg total	.61^***^	−.15^***^	.48^***^	.60^***^	.51^***^	46.72	17.62
	IV) motivation	0.06	.68^***^	.19^***^	.08^*^	0.05	10.78	5.02
	V) analytic thinking	.18^***^	.63^***^	.30^***^	.23^***^	.14^**^	14.88	5.60
	COWS-pos total	.14^**^	.72^***^	.27^***^	.18^***^	.10^*^	25.66	9.69
	MCQ-neg	–	.11^**^	.76^***^	.77^***^	.76^***^	11.55	4.37
	MCQ-pos		–	.27^***^	.13^**^	.08^*^	10.74	3.80
	PSWQ			–	.67^***^	.68^***^	40.66	7.86
	PTQ				–	.69^***^	28.87	12.80
	GAD-Q-IV					–	3.78	3.77

**Notes.**

^∗^*p* < .05, ^∗∗^*p* < .01, ^∗∗∗^*p* < .001 (two-sided).

WW II Why Worry-II (total score) COWS-neg COWS I, II, III COWS-pos COWS IV, V MCQ-neg Metacognitions Questionnaire factor 1 MCQ-pos Metacognitions Questionnaire factor 2 PSWQ Penn State Worry Questionnaire PTQ Perseverative Thinking Questionnaire GAD IV Generalized Anxiety Disorder Questionnaire-IV

#### Convergence

As expected, the subscales measuring positive beliefs (WW-II subscales I-V, COWS subscales IV & V) showed significant medium to strong correlative relationships with each other (.44 < *r*’s  <.76; all *p*’s < .001) and with the MCQ-30 subscale describing positive metacognitions (WW-II total scale: *r* = .74, *p* < .001; COWS-pos: *r* = .72, *p* < .001).

Likewise, the subscales capturing negative beliefs (COWS subscales I - III) showed significant and substantial inter-correlations (.73 < *r*’s  <82; all *p*’s < .001) and a strong correlation with the MCQ-30 factor about negative metacognitive beliefs (*r* = .61, *p* < .001).

#### Divergence

As hypothesized, subscales measuring positive metacognitions showed generally low and mostly non-significant associations with those capturing negative beliefs (−.26 < *r*’s < .21). With regard to MCQ-neg, WW-II total score (*r* = .27) and COWS-pos (*r* = .14) likewise revealed small correlations. The same applies for COWS-neg and the relationship with MCQ-pos (*r* =  − .15).

### Predictive value of the measures

#### WWII and COWS predicting worry, RNT and GAD symptoms

To test whether positive and negative metacognitions substantially contribute to predicting worry, repetitive negative thinking, and GAD symptoms, respectively, stepwise multiple regression analyses were conducted with PSWQ, PTQ, and GAD-Q-IV scores as dependent variables and the Why Worry-II and COWS subscales as predictor variables.

In Step 1, all five subscales of the WW-II assessing positive metacognitions were included. In Step 2, the COWS subscales measuring positive metacognitions (COWS-pos) were entered. In a final Step 3, the COWS subscales referring to negative beliefs (COWS-neg) were included as additional predictors. To check for multicollinearity, all variables were inspected with regard to their specific tolerance level (which for all variables did not show below the cut-off .25 recommended by Urban & Mayerl, 2006) and Variance Inflation Factor (VIF; which for all variables did not show above the cut-off 5.0 recommended by Urban & Mayerl, 2006).

As displayed in Table 6, of all subscales measuring positive metacognitions, Why Worry-II scale III (“worry protects against negative emotions”) showed the closest relationship to all three dependent variables (*Step 2:* .33 < *β* < .39; all *p*’s < .001). Furthermore it still remained as a significant predictor in the model (*Step 3:* .12 < *β* < .23; all *p*’s < .001), negative metacognitions (COWS-neg: COWS subscales I, II, III) were added.

**Table 6:** Summary of stepwise multiple regression analyses of PSWQ, PTQ and GAD-Q-IV: *β* coefficients (SE B).

Independent variables		PSWQ			PTQ			GAD-Q-IV		
		*Step 1*	*Step 2*	*Step 3*	*Step 1*	*Step 2*	*Step 3*	*Step 1*	*Step 2*	*Step 3*
WW-II	I) problem solving	−.08 (.07)	−.11 (.07)	−.03 (.06)	−.01 (.07)	−.03 (.07)	.07 (.06)	−.10 (.07)	−.08 (.07)	.01 (.06)
	II) motivation	−.02 (.06)	.00 (.06)	.09 (.06)	−.18^**^ (.06)	−.13^*^ (.06)	−.02 (.05)	−.15^*^ (.06)	−.10^*^ (.07)	−.04 (.06)
	III) protection	.41^***^ (.05)	.39^***^ (.05)	.23^***^ (.05)	.35^***^ (.06)	.33^***^ (.06)	.12^**^ (.04)	.33^***^ (.06)	.33^***^ (.06)	.15^**^ (.05)
	IV) prevention	.01 (.06)	.03 (.06)	.01 (.05)	.06 (.06)	.08 (.06)	.06 (.05)	.04 (.06)	.04 (.06)	.03 (.05)
	V) positive trait	.14^*^ (.06)	.13^*^ (.06)	.09 (.05)	.13^*^ (.06)	.11 (.06)	.07 (.05)	.19^**^ (.06)	.18^**^ (.06)	.16^**^ (.05)
COWS-pos	IV) motivation		−.07 (.06)	−.02 (.05)		−.15^*^ (.06)	−.08 (.05)		−.10 (.06)	−.04 (.05)
	V) analytic thinking		.13^*^ (.05)	.09 (.05)		.15^**^ (.06)	.10^*^ (.04)		.02 (.06)	−.04 (.05)
COWS-neg	I) disruption			.03 (.06)			.01 (.06)			−.10 (.06)
	II) problem exaggeration			.28^***^ (.06)			.37^***^ (.06)			.27^***^ (.06)
	III) emotional discomfort			.20^***^ (.05)			.26^***^ (.05)			.36^***^ (.05)
	*df*	5, 597	7, 595	10, 592	5, 597	7, 595	10, 592	5, 597	7, 595	10, 592
	*F* change	32.93^***^	3.13^*^	69.51^***^	24.18^***^	4.92^***^	128.55^***^	20.25^***^	1.41	75.81^***^
	*adjusted R^2^*	.21	.22	.42	.16	.17	.50	.14	.14	.38
	*R^2^ change*	.21	.01	.20	.16	.01	.33	.14	.004	.24

**Notes.**

^∗^*p* < .05, ^∗∗^*p* < .01, ^∗∗∗^*p* < .001.

Criterion and predictor variables are *z*-standardized - *β* coefficients are therefore equivalent to B and displayed only.

Two scales assessing negative metacognitive beliefs (COWS factor II “worrying exaggerates the problem” with .27 < *β* < .37; all *p*’s < .001 and factor III “worrying causes emotional discomfort” with .20 < *β* < .36; all *p*’s < .001) explained the greatest amount of variance in the final model.

When focusing on repetitive negative thinking (PTQ) as the dependent variable, COWS subscale IV (“worry helps analytic thinking”) additionally made a unique contribution to variance explanation (*Step 3: β* = .10, *p* = .03). As for the GAD-Q-IV, Why Worry-II factor V (“worry reflects a positive personality trait”) additionally contributed to the prediction of GAD symptoms at a statistically significant level (*step 3: β* = .16, *p* = < .01).

#### MCQ-30, WW-II and COWS predicting worry, RNT and GAD symptoms

In a final set of regression analyses, we tested whether WW-II and COWS still predicted worry, repetitive negative thinking and GAD symptoms, when the MCQ-30 as the gold standard measure of metacognitions is controlled.

Predicting worry (PSWQ): When adding MCQ-pos (Table 7, Step 1) and afterwards the WW-II scales as well as both COWS subscales referring to positive beliefs about worry, the prediction is optimized (Step 2), while it can be further improved by also using MCQ-neg (Step 3). However, when entering all three COWS subscales referring to negative beliefs about worry, variance explanation is not improved (Step 4).

**Table 7:** Summary of stepwise multiple regression analyses of PSWQ, PTQ and GAD-Q-IV in addition to the MCQ-30: *β* coefficients (SE B).

		PSWQ				PTQ				GAD-Q-IV			
*IVs*		*Step 1*	*Step 2*	*Step 3*	*Step 4*	*Step 1*	*Step 2*	*Step 3*	*Step 4*	*Step 1*	*Step 2*	*Step 3*	*Step 4*
*MCQ-pos*		.27^***^(.04)	.11^*^ (.06)	.15^**^ (.05)	.16^***^(.05)	.13^**^(.04)	−.11 (.07)	−.06 (.05)	−.04 (.04)	.08 (.04)	−.11 (.07)	−.06 (.05)	−.06 (.05)
WW-II	I)		−.17^*^ (.08)	−.13^*^ (.06)	−.12^*^ (.06)		.03 (.08)	.08 (.06)	.09 (.05)		−.01 (.08)	.04 (.06)	.04 (.06)
	II)		.00 (.06)	.10^*^ (.04)	.11^*^ (.05)		−.13 (.06)	−.02 (.05)	.00 (.04)		−.10 (.07)	.01 (.05)	−.01 (.05)
	III)		.39^***^(.05)	.13^**^ (.04)	.12^**^ (.04)		.33^***^(.06)	.06 (.04)	.03 (.04)		.33^***^(.06)	.05 (.04)	.04 (.04)
	IV)		.03 (.06)	−.04 (.04)	−.03 (.04)		.07 (.06)	.01 (.04)	.02 (.04)		.04 (.06)	−.03 (.04)	−.02 (.04)
	V)		.12^*^ (.06)	.04 (.04)	.04 (.04)		.12 (.06)	.03 (.04)	.03 (.04)		.19^**^ (.06)	.10^*^ (.04)	.11^*^ (.04)
COWS-pos	IV)		−.10 (.06)	−.04 (.04)	−.03 (.04)		−.12 (.06)	−.06 (.04)	−.05 (.04)		−.08 (.06)	−.02 (.04)	−.01 (.04)
	V)		.13^*^ (.05)	.07 (.04)	.06 (.04)		.16^**^ (.06)	.10^*^ (.04)	.08 (.04)		.03 (.06)	−.04 (.04)	−.05 (.04)
*MCQ-neg*				.70^***^(.03)	.63^***^(.04)			.72^***^(.03)	.54^***^(.04)			.74^***^(.03)	.67^***^(.04)
COWS-neg	I)				.06 (.05)				.03 (.05)				−.07 (.05)
	II)				.02 (.05)				.15^**^ (.05)				−.01 (.05)
	III) discomfort				.04 (.04)				.11^**^ (.04)				.18^***^(.04)
*df*		1, 601	8, 594	9, 593	12, 590	1, 601	8, 594	9, 593	12, 590	1, 601	8, 594	9, 593	12, 590
*F* change		47.677^***^	16.961^***^	616.621^***^	3.541^*^	10.475^*^	17.544^***^	644.206^***^	21.822^***^	4.17^*^	14.594^***^	640.659^***^	6.1^***^
*Adj. R^2^*		.07	.22	.62	.62	.02	.18	.60	.64	.01	.14	.59	.60
*R^2^ change*		.07	.15	.40	.01	.02	.17	.42	.04	.01	.15	.44	.01


**Notes.**

^∗^*p* < .05, ^∗∗^*p* < .01, ^∗∗∗^*p* < .001.

*IV*, Independent Variable; criterion and predictor variables are *z*-standardized - *β* coefficients, B and displayed only.

*Predicting negative repetitive thinking (PTQ):* When entering MCQ-pos (Step 1) and all five WW-II subscales as well as the two COWS subscales referring to positive beliefs about worry afterwards (Step 2), the prediction once again is optimized while it can likewise be further improved by using the MCQ-neg as well (Step 3). This time, variance explanation can be improved, by adding the three COWS subscales referring to negative beliefs about worry (Step 4), which causes all factors with positive beliefs to no longer substantially contribute to variance explanation though (see Table 7).

*Predicting GAD symptoms (GAD-Q-IV):* First, when adding MCQ-pos (Step 1), no significant amount of variance explanation can be detected, whereas adding the WW-II scales in addition to the COWS scales afterwards (Step 2), leads to a significant amount of explained variance, which again can be substantially improved by entering MCQ-neg (Step 3) and also by including all three COWS subscales referring to negative beliefs about worry (Step 4).

## Discussion

The main purpose of this study was to validate German versions of the WW-II and COWS as questionnaire measures of metacognitions related to worry. Our findings suggest that both questionnaires possess good psychometric properties that are comparable to the original English versions. This includes high internal consistency for all subscales as well as correlational patterns with other questionnaire measures supporting convergent and divergent validity.

In addition, the dimensional structure identified for the original questionnaires and predicted by theory, showed good fit with our data for the German versions. For both questionnaires, the proposed five-factor structures showed good fit. All items significantly loaded onto their respective subscale, and each subscale showed good internal consistency. In sum, our findings suggest that WW-II and COWS can be used as reliable and valid measures of metacognitions related to worry.

Although the main aim of our study was the validation of questionnaire measures, we think that the results of the regression analyses are also informative from a theoretical point of view, with regard to the role of metacognitions, specific contributions of negative and positive metacognitions and possible subtypes in the latter case. First, about 50% of the variance in worry, GAD symptoms and repetitive negative thinking could be statistically explained by metacognitive beliefs. This supports the idea put forward by cognitive models of GAD that metacognitive beliefs are crucial in initiating and maintaining worrisome thinking.

Second, the relative contributions of the different metacognition subscales to predicting symptom levels is of interest. Generally, our findings are in line with previous studies’ outcomes (i.e.; Thielsch, Andor & Ehring, 2015a; Thielsch, Andor & Ehring, 2015b), revealing negative metacognitions as comparatively more closely linked to worry than positive metacognitions. The same pattern was found when focusing on negative repetitive thinking or GAD symptoms as alternative dependent variables, which underlines the robustness of the finding. Past research using the MCQ-30 has largely focused on the negative metacognitive belief related to uncontrollability of worrisome thinking, showing close relationships between this specific negative metacognitions and worry. Likewise, our study shows a close relationship between this specific MCQ-30-scale and all worry measures. Our findings suggest that additional facets of negative metacognitions may also be important, such as represented in the COWS subscales “worrying exaggerates the problem” and “worrying causes emotional discomfort”. Interestingly, these two subscales explained additional variance over and above the MCQ-30 for the dependent variables RNT and GAD symptoms, but not for the PSWQ. However, as described in the introduction it cannot be ruled out that the strong association between the MCQ-30 and the PSWQ may at least partly be due to criterion contamination as both measures assess uncontrollability of thinking in a similar way (example item of the MCQ-30 scale: “When I start worrying I cannot stop.”; example item of the PSWQ: “Once I start worrying, I cannot stop.”).

Third, the MCQ-30 positive metacognitions subscale mainly focuses on beliefs related to worrying as a problem-solving strategy (e.g., “Worrying helps me to get things sorted out in my mind.”, “I need to worry in order to remain organized.”, “Worrying helps me to avoid problems in the future.”). Our findings suggest that additional subtypes of positive metacognitions need to be additionally considered. Among all subscales addressing positive beliefs, the WW-II subscale “worry protects against negative emotions” showed the highest and most stable predictive value for all three dependent variables, and as far as the PSWQ is concerned even when controlling for negative metacognitive beliefs. This is in line with theoretical models of GAD suggesting that worry serves to avoid the experience of negative emotions (i.e., Borkovec & Roemer, 1995; Newman et al., 2014). In sum, the relationship between positive metacognitions and worry might be underestimated by using the gold standard MCQ-30 only, as this represents only a restricted operationalization of the concept while not capturing the theoretically postulated subtypes (Dugas & Robichaud, 2012). This consideration is supported by this study’s results, showing a substantial improvement of variance explanation due to COWS-positive and WW-II scales over and above the MCQ-30 subscale.

Taken all this together, our findings support the idea that metacognitions related to worry should be assessed using multiple questionnaire measures. In order to provide a more fine-grained analysis of positive metacognitions, the Why Worry Scale - II appears well suited to be given in addition to the MCQ-30. The COWS, on the other hand, has been shown to be a useful measure of negative metacognitions and can be regarded as an interesting addition to the MCQ-30 assessment. As the MCQ-30 is a well-validated measure for metacognitions itself, the questionnaires translated in this study therefore serve as a supplement and as amplification whenever a more fine-grained measurement is needed.

Some limitations are noteworthy when interpreting the results. Most importantly, we evaluated the questionnaires in a non-clinical sample. This is defendable considering the nature of worry as a cognitive phenomenon being conceptualized on a continuum with similar underlying mechanisms in pathological and non-pathological worry (Tallis, Eysenck & Mathews, 1992). Nevertheless, the use of the questionnaires in measuring pathological worry should be further investigated. In addition, it remains to be shown whether results from our online assessment can be replicated using other types of assessment. Reassuringly, however, earlier research has shown that results of web-based assessments are equivalent to traditional assessment format (Naus, Philipp & Samsi, 2009; Skitka & Sargis, 2006).

## Conclusions

Our findings support the idea that metacognitive beliefs are important in the development and/or maintenance of excessive worry and metacognitions should best be assessed using multiple measures assessing different aspects of these constructs. The German versions of the Why Worry Scale-II and the Consequences of Worry Scale can be recommended to be used in addition to the gold standard Metacognitions Questionnaire-30 (MCQ-30). Our results suggest that they are reliable and valid self-report measures for assessing different facets of metacognitive beliefs.

##  Supplemental Information

10.7717/peerj.5177/supp-1 Data S1 Raw data file (SPSS)

## Additional Information and Declarations

### Data Availability

The following information was supplied regarding data availability: The raw data are provided in Data S1.
